# Correction: Compensatory induction of MYC expression by sustained CDK9 inhibition via a BRD4-dependent mechanism

**DOI:** 10.7554/eLife.09993

**Published:** 2015-07-17

**Authors:** Huasong Lu, Yuhua Xue, Guoying K Yu, Carolina Arias, Julie Lin, Susan Fong, Michel Faure, Ben Weisburd, Xiaodan Ji, Alexandre Mercier, James Sutton, Kunxin Luo, Zhenhai Gao, Qiang Zhou

Lu H, Xue Y, Yu GK, Arias C, Lin J, Fong S, Faure M, Weisburd B, Xiaodan J, Mercier A, Sutton J, Luo K, Gao Z, Zhou Q. 2015. Compensatory induction of MYC expression by sustained CDK9 inhibition via a BRD4-dependent mechanism. *eLife* **4**:e06535. doi: 10.7554/eLife.06535

Published 17 June 2015

In the published article, author Yuhua Xue was misspelt as ‘Yuahua Xue’.

This article has been corrected accordingly.

